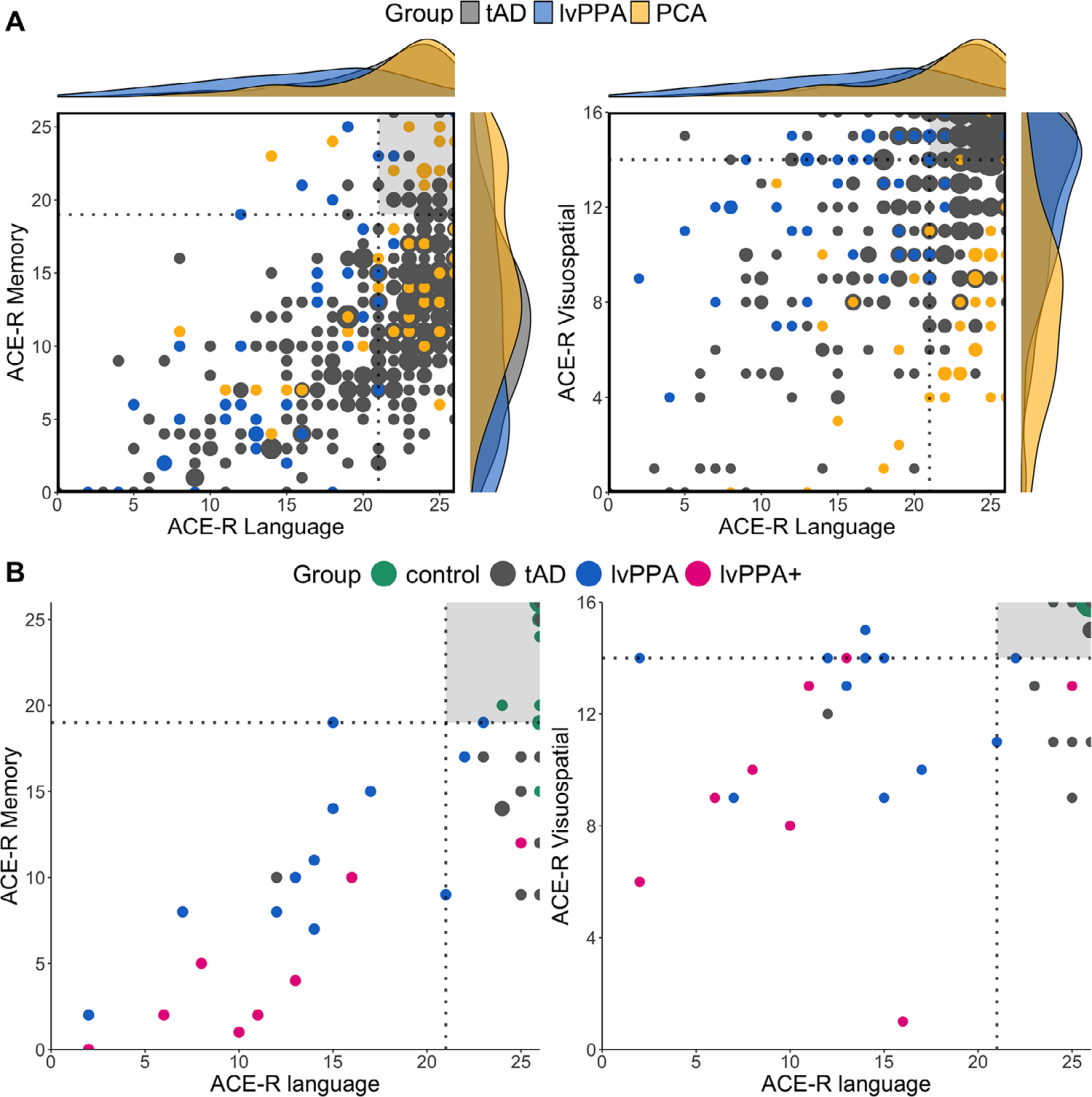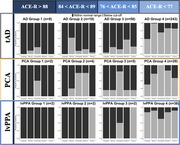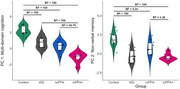# Multidimensional cognitive deficits in the typical and atypical variants of Alzheimer's disease

**DOI:** 10.1002/alz70857_103552

**Published:** 2025-12-25

**Authors:** Shalom K Henderson, Alexander G Murley, Thomas E Cope, Lucy Bowns, Maura Malpetti, Karalyn E Patterson, James B Rowe, Matthew A Lambon Ralph

**Affiliations:** ^1^ University of Cambridge, Cambridge, United Kingdom; ^2^ Unviersity of Cambridge, Cambridge, United Kingdom; ^3^ University of Cambridge, Cambridge, ‐, United Kingdom

## Abstract

**Background:**

There is ongoing debate about whether Alzheimer's disease (AD) phenotypes are distinct clinical entities or represent positions within a graded multidimensional space. There are confounding factors that make it difficult to draw definitive conclusions between these two hypotheses which we addressed in the present study.

**Method:**

In this two‐part investigation, we first examined the comparative distributions of cognitive performance in people diagnosed with typical amnestic AD (tAD), logopenic variant of primary progressive aphasia (lvPPA), and posterior cortical atrophy (PCA) using a large retrospective dataset of past research participants (*n* = 413) from memory clinics. Secondly, a prospective deep phenotyping study of lvPPA (*n* = 18) compared to typical AD (*n* = 9) addressed the questions regarding the severity of patients, granularity of testing, nature of the assessment, and differences in cross‐sectional versus longitudinal study design. In addition, we explored the associations between scores derived from a principal component analysis on cognitive measures and grey matter volumes in key memory‐ and language‐related brain regions, at baseline and longitudinally.

**Result:**

Both the prospective and retrospective data revealed: (i) patients showing graded distinctions (e.g., predominant visual versus language impairment in people with PCA versus lvPPA) and overlap (e.g., shared weakness in domains such as memory); and (ii) people with lvPPA and tAD being equally impaired in episodic memory. Moreover, the deep phenotyping study showed similar patterns of deficits in verbal and non‐verbal memory tests in lvPPA and tAD patients. Longitudinal assessment revealed: (i) people with tAD showing varied patterns of phenotypic differentiation; and (ii) people with lvPPA and lvPPA+ exhibiting a multidimensional pattern of decline. The results of Bayesian linear regressions showed evidence for the association of grey matter volumes in language and memory networks, including the bilateral hippocampi, precuneus, posterior cingulate, and temporo‐parietal regions with principal component analysis derived scores.

**Conclusion:**

The graded distinctions amongst typical amnestic and atypical phenotypes of AD support the proposal for a transdiagnostic, multidimensional phenotype geometry that spans all AD subtypes. Inclusion of all AD phenotypes in clinical trials and treatments is critical with a particular focus on transdiagnostic symptoms, considering their relevance to disease burden and interventions.